# Sperm and Spermatids Contain Different Proteins and Bind Distinct Egg Factors

**DOI:** 10.3390/ijms150916719

**Published:** 2014-09-19

**Authors:** Marta Teperek, Kei Miyamoto, Angela Simeone, Renata Feret, Michael J. Deery, John B. Gurdon, Jerome Jullien

**Affiliations:** 1 Department of Zoology, University of Cambridge, Downing Street, Cambridge CB2 3EJ, UK; E-Mails: mt446@cam.ac.uk (M.T.); k.miyamoto@gurdon.cam.ac.uk (K.M.); angela.simeone@gurdon.cam.ac.uk (A.S.); j.gurdon@gurdon.cam.ac.uk (J.B.G.); 2 Wellcome Trust/Cancer Research UK Gurdon Institute, University of Cambridge, Tennis Court Road, Cambridge CB2 1QN, UK; 3 Cambridge Centre for Proteomics, University of Cambridge, Tennis Court Road, Cambridge CB2 1QR, UK; E-Mails: rf280@cam.ac.uk (R.F.); md289@cam.ac.uk (M.J.D.)

**Keywords:** sperm, spermatid, egg extract, proteome, chromatin, mass spectrometry, spermiogenesis, chromatin remodelers, transcription, programming

## Abstract

Spermatozoa are more efficient at supporting normal embryonic development than spermatids, their immature, immediate precursors. This suggests that the sperm acquires the ability to support embryonic development during spermiogenesis (spermatid to sperm maturation). Here, using *Xenopus laevis* as a model organism, we performed 2-D Fluorescence Difference Gel Electrophoresis (2D-DIGE) and mass spectrometry analysis of differentially expressed proteins between sperm and spermatids in order to identify factors that could be responsible for the efficiency of the sperm to support embryonic development. Furthermore, benefiting from the availability of egg extracts in *Xenopus*, we also tested whether the chromatin of sperm could attract different egg factors compared to the chromatin of spermatids. Our analysis identified: (1) several proteins which were present exclusively in sperm; but not in spermatid nuclei and (2) numerous egg proteins binding to the sperm (but not to the spermatid chromatin) after incubation in egg extracts. Amongst these factors we identified many chromatin-associated proteins and transcriptional repressors. Presence of transcriptional repressors binding specifically to sperm chromatin could suggest its preparation for the early embryonic cell cycles, during which no transcription is observed and suggests that sperm chromatin has a unique protein composition, which facilitates the recruitment of egg chromatin remodelling factors. It is therefore likely that the acquisition of these sperm-specific factors during spermiogenesis makes the sperm chromatin suitable to interact with the maternal factors and, as a consequence, to support efficient embryonic development.

## 1. Introduction

Spermatogenesis is the process which leads to the creation of mature sperm. It consists of two stages: meiotic maturation and spermiogenesis. During meiotic maturation sperm precursors reduce their DNA and chromosome content. After the completion of meiotic division they are called spermatids. Spermatids are therefore the first precursors of sperm with haploid chromosome content. However, in order to transform into fully-differentiated sperm, spermatids have to complete the second stage of spermatogenesis: spermiogenesis. Spermiogenesis is a complex, multistep process, involving numerous molecular and morphological changes in the maturing cells. This is manifested by the fact that round spermatids are morphologically similar to somatic cells, whereas mature spermatozoa are highly specialized, with a compacted DNA contained in the sperm head and a long tail, ensuring motility at fertilization [1]. Since dramatic changes occur during spermiogenesis, it is not surprising that this process is accompanied by the loss and gain of many proteins [2,3,4,5,6].

Interestingly, experiments in mouse in which embryos were generated by injecting either sperm or spermatids into unfertilized oocytes, showed that sperm are better at supporting embryonic development than spermatids [7]. This suggests that the ability of sperm to support efficient embryonic development is acquired during spermiogenesis. It is therefore possible that the loss or gain of particular proteins in the course of spermiogenesis is responsible for the acquisition of the developmental advantage of sperm, as compared to spermatids. Such proteins could have a direct or indirect effect on embryonic development. For example, if sperm delivered transcription factors to the embryo, these transcription factors could directly influence the embryonic development by instructing the embryo which genes to express [4,8]. On the other hand, sperm-specific factors could also have an indirect effect on development if they were recognized by egg-derived effector proteins. For example, sperm-derived protamines are recognized and processed by egg-derived nucleoplasmin, which helps the sperm nucleus to rearrange its DNA wrapping [9,10] and to acquire a chromatin state compatible with early development.

In this work we aimed to characterize proteins that change during spermiogenesis with the goal of finding candidate factors that could explain the difference in the developmental potential between sperm and spermatids. To address this question we first compared proteins present in sperm and spermatids. In order to get insight into possible interactions between sperm/spermatids and the egg, we also investigated what egg factors bind specifically to the chromatin of sperm or spermatids. To facilitate the analysis, we chose *Xenopus laevis* as a model organism. We benefited from the fact that egg extracts, which can recapitulate the whole first embryonic cell cycle [11,12] are easily available in frogs. Here we incubated sperm and spermatids in egg extracts to investigate the binding of egg factors onto sperm/spermatid chromatin. Proteins differentially expressed between sperm and spermatids, or differentially bound to sperm and spermatids chromatin were visualized by 2D-DIGE electrophoresis (2-D Difference In-Gel Electrophoresis) and identified by Liquid chromatography-mass spectrometry (LC-MS/MS) mass spectrometry analysis. In summary, our approach led to the identification of 51 sperm-specific proteins and 47 spermatid-specific proteins. Additionally, we also identified 107 egg proteins binding specifically to sperm chromatin upon egg-extract treatment, and 20 egg proteins incorporated specifically into spermatid chromatin upon egg extract incubation. Many factors identified specifically in sperm, or incorporated from eggs into the sperm chromatin, are important for the maintenance of chromatin structure and the regulation of transcription (e.g., Mbd3, Hp1γ, Wdr5, Mlf1). These results therefore suggest that the acquisition of a specific set of proteins in sperm could indeed reflect its programming to support embryonic development.

## 2. Results and Discussion

### 2.1. Sperm and Spermatids Differ in Their Nuclear Protein Composition

#### 2.1.1. Sperm and Spermatids Contain Different Proteins

**Figure 1:**
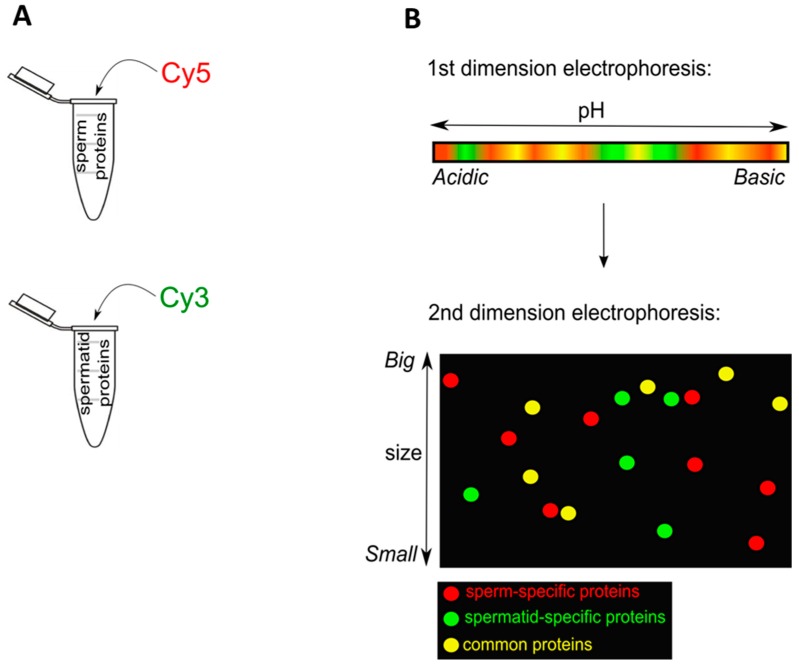
Experimental design for 2-D Fluorescence Difference Gel Electrophoresis (2D-DIGE) analysis. (**A**) The same quantity of proteins isolated from sperm or spermatids was labelled with Cy5 (red) or Cy3 (green) dye; (**B**) After labelling, proteins were mixed and separated in the first dimension electrophoresis in the pH range, according to isoelectric points of proteins. Subsequently, the proteins were run in the second dimension electrophoresis, according to the molecular weight of proteins. These two runs allowed separation of proteins into spots of three colours: in this example the red spots were sperm-specific; the green ones spermatid-specific and the yellow ones were proteins present in both cell types.

**Figure 2:**
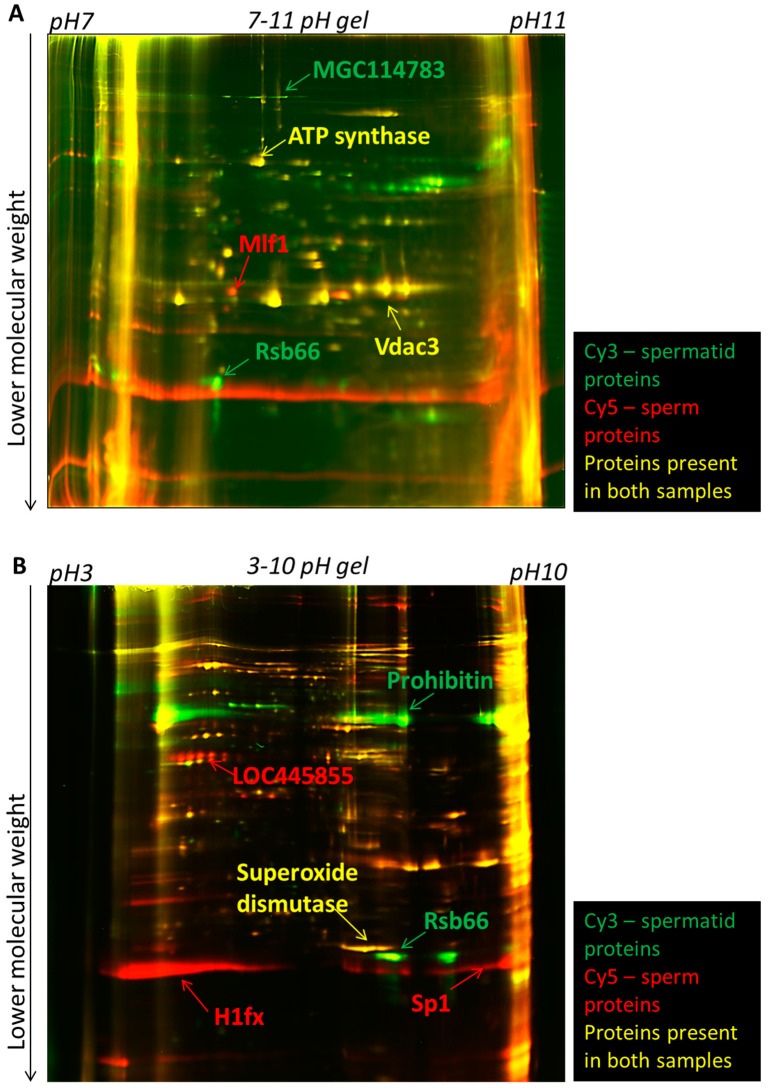
2D-DIGE electrophoresis of proteins isolated from sperm and spermatids. Proteins isolated from spermatids and sperm were labelled with Cy3 (green) and Cy5 (red) dyes, respectively, and subsequently separated in two dimensions. Examples of sperm- and spermatid-specific proteins identified are indicated with arrows: red arrows for sperm-specific proteins and green arrows for spermatid-specific proteins. (**A**) Laser scanned image of a gel with the proteins separated in pH range 7–11 (basic range); (**B**) Laser scanned image of gel with proteins separated in the pH range 3–10 (broad range).

**Figure 3:**
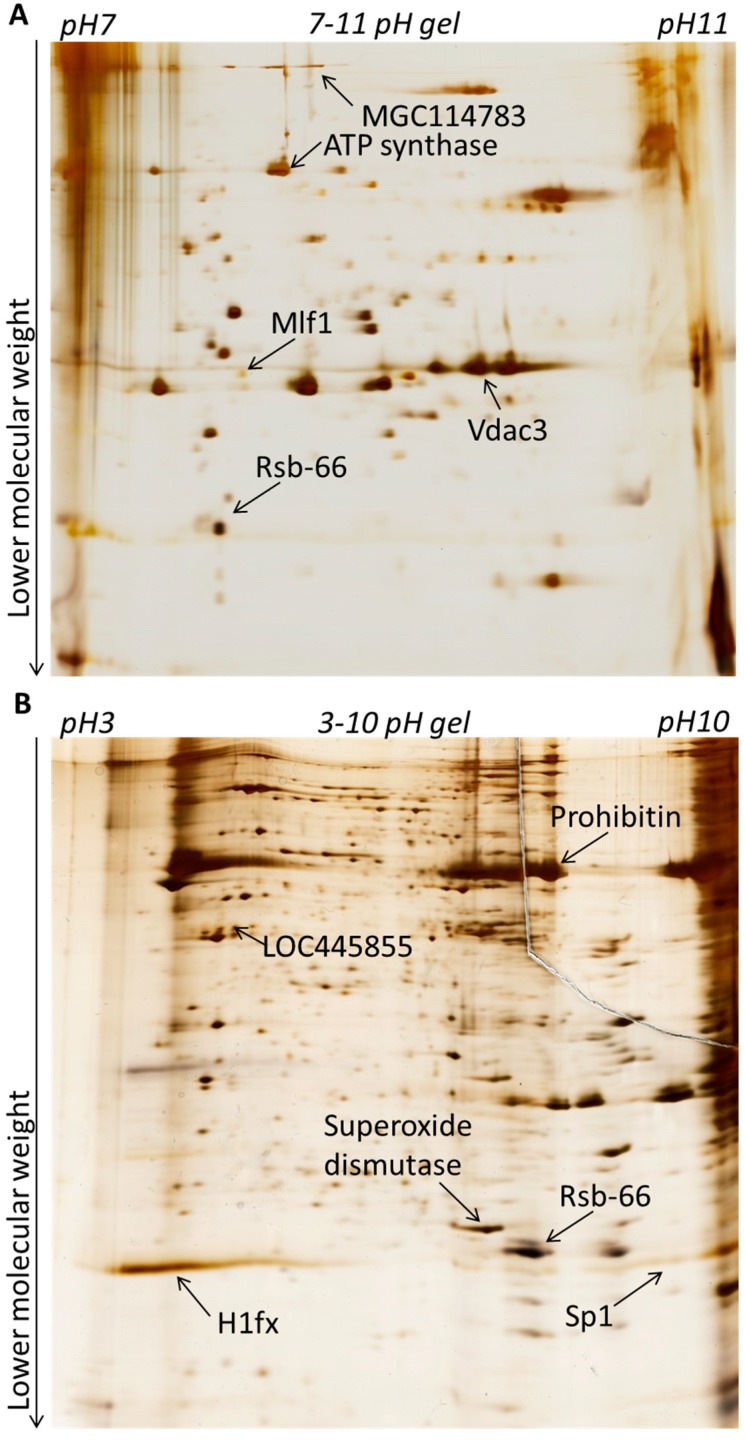
Silver staining of 2D-DIGE gels for spot excision. Gels from Figure 2 were silver-stained to allow protein visualisation and spot excision. Protein examples from Figure 2 are also indicated with arrows. (**A**) proteins separated in pH 7–11; (**B**) proteins separated in pH 3–10.

We first investigated differences in nuclear composition between sperm and spermatids. Sperm and spermatids were purified by density gradient centrifugation as described before [13] with minor modifications [14] (see Experimental Section). Proteins were extracted from permeabilized sperm and spermatid with Urea/Thiourea buffer (see Experimental Section). Subsequently, equal amount of proteins isolated from sperm and spermatids were labelled with fluorescent Cy5 and Cy3 dyes, respectively, and separated in two dimensions (Figure 1A,B). It is known that many proteins, which become incorporated into the sperm chromatin in *Xenopus laevis*, are highly basic [2,15,16]. For example, one of the most abundant proteins constituting the nucleus of mature sperm in *Xenopus laevis* are sperm basic proteins. Six sperm basic proteins were identified and named Sp1–Sp6, for Sperm basic protein 1–6 [15]. These proteins do not share sequence conservation to mammalian protamines; however, they are thought to be their functional orthologues, since similarly to mammalian protamines, they are more basic than histones and are implicated in compacting the nucleus in mature sperm [15]. Therefore, in order to allow an appropriate separation of all proteins and of basic nuclear proteins, the first dimension electrophoresis (separating proteins according to their isoelectric point) was performed separately in two different pH ranges: either pH 3–10 (to better separate the majority of the proteins) or pH 7–11 (to specifically separate the basic proteins). Subsequently, all the proteins were separated by electrophoresis in the second dimension, according to their molecular mass. Gels were imaged using a laser scanner to manually identify qualitative differences in the protein spots presence. We identified spots present specifically in sperm, spermatids and also spots which were common between the two samples (Figure 2A,B). Afterwards, gels were silver-stained in order to allow visualisation and excision of selected protein spots (Figure 3A,B). Subsequently, proteins isolated from each spot were subjected to mass spectrometry analysis. Only those proteins which had an overall Mascot protein probability score above 100, or a score below 100 [17], but at least two different peptides confirming their identity, were included in the final list of identified proteins. In total, 51 sperm-specific, 47 spermatid-specific and 38 proteins present in both cell types were identified (Table S1). Amongst the sperm-specific proteins identified, proteins previously reported to be present in *Xenopus laevis* sperm were found, for example sperm basic protein 1 (Sp1), sperm basic protein 4 (Sp4) (Sp1–6 proteins are functional orthologues of mammalian protamines) and histone H1 variant H1fx [18]. Similarly, in the list of spermatid-specific proteins a homologue of a human spermatid-specific protein, Rsb-66, was found [19,20]. Proteins that were found in both cell types contained mainly proteins involved in metabolism and also in the organization of cytoskeleton, for example actin, tubulin or ATP synthase (Table S1).

#### 2.1.2. Sperm-Specific Proteins Contain Several Factors Which May Support Subsequent Embryonic Development

Within the sperm-specific proteins there are several which could be important for embryonic development after fertilization. For example, we have identified Wdr5 (beta-transducin repeats (WD) domain 5) specifically in sperm nuclei. Wdr5 recognizes and binds to dimethylated and trimethylated lysine 4 of histone H3 (H3K4me2/3) and recruits histone H3K4 methylase (via a direct interaction with the methylase), which results in further spreading of the activating H3K4me2/3 epigenetic mark. Such spreading of this mark ultimately leads to transcriptional activation [21,22,23]. Knockdown of Wdr5 in *Xenopus laevis* embryos leads to abnormal expression of developmentally important *Hox* genes. Furthermore, Wdr5 was shown to be required for the self-renewal of embryonic stem cells (ES cells) and also for the establishment of pluripotency during the derivation of induced pluripotent stem cells (iPS cells) [24]. Therefore, the presence of Wdr5 protein in the sperm, but not in the spermatid nucleus, could be advantageous for the sperm to support development, as Wdr5 protein is directly involved in the regulation of transcription.

Another example of a sperm-specific protein identified in this study is Mlf1 (Myeloid leukemia factor 1). Mlf1 is a transcription factor that regulates both gene transcription and cell cycle progression [25,26,27]. In *Xenopus* early embryonic development, cell cycle phases are very rapid, therefore the presence of Mlf1 could be important for supporting these rapid cell cycles at the earliest stages of embryonic development, and could potentially explain the developmental advantage of sperm over the spermatids.

Conversely, proteins named Prohibitin and Prohibitin 2 were identified as being present specifically in spermatids. They are highly conserved, with 90% and 88% amino acids identity between *Mus musculus* and *Xenopus laevis*, respectively. They have anti-proliferative functions, and they are also involved in differentiation and morphogenesis [28]. It is thus possible that the presence of certain proteins in the spermatids, but not in sperm, such as anti-proliferative Prohibitins, could impair the development of spermatid-derived embryos.

#### 2.1.3. Chromatin and Nuclear Proteins Are Enriched in Sperm

We also tested whether any particular types of proteins are enriched in sperm and spermatids. For this purpose we performed gene ontology (GO) analysis using the online Database for Annotation, Visualization and Integrated Discovery (DAVID) [29,30]. Gene ontology analysis indeed indicated that distinct classes of proteins are overrepresented in sperm and spermatids (*p*-value < 0.05, Table S2). GO analysis of biological processes (BP) for sperm-specific proteins showed a significant enrichment for terms associated with chromatin and nuclear changes, for example nucleosome organisation, nucleosome assembly or DNA packaging. This likely reflects the high degree of sperm nucleus specialisation that occurs during spermiogenesis (incorporation of protamines and global chromatin remodelling). In agreement with that, such terms were not enriched in the list of spermatid proteins. Among spermatid proteins the only significantly enriched BP terms were related to cellular homeostasis. Similarly, the BP terms enriched among the proteins present in both cell types were related to basic metabolism, such as glycolysis, oxidation reduction or anion transport (Table S2).

### 2.2. Sperm and Spermatids Attract Distinct Egg Factors

#### 2.2.1. Dramatic Changes in Protein Composition after the Egg Extract Treatment

We next tested whether differences in the developmental potential between sperm and spermatids could be also reflected by their differential ability to attract and bind egg factors after fertilisation. One cell embryos contain only one set of maternal and paternal chromosomes, making the analysis of chromatin-bound proteins challenging for two reasons. First, there is an equimolar amount of paternal and maternal chromosomes, which does not allow the analysis of the factors bound only to the paternal chromosomes. Second, just one set of chromosomes per embryo limits the material availability. To overcome these limitations, we took advantage of the availability of egg extracts in *Xenopus laevis*. Extracts from activated eggs can recapitulate the whole first embryonic cell cycle: global protamine to histone exchange in the sperm nucleus, DNA synthesis and chromosome condensation for mitosis [11,12]. Incubating a large number of sperm/spermatids in extracts prepared from activated eggs overcomes the problem of a limited amount of material in 1-cell stage embryos and mimics the events happening after fertilisation. Therefore, to test whether sperm and spermatids attract different egg factors, we incubated permeabilised sperm and spermatids in egg extracts for 1 h, time sufficient for pronuclei formation and replication initiation [11].

**Figure 4:**
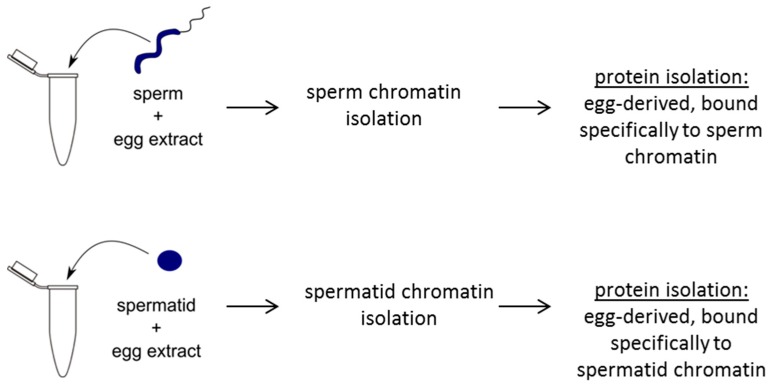
Schematic diagram showing experimental design for mass spectrometry analysis of extract-treated sperm or spermatids. Sperm or spermatids are separately treated with egg extracts. Subsequently, sperm or spermatid chromatin is purified and chromatin-bound proteins are isolated. Isolated proteins are then subjected to 2D-DIGE and mass spectrometry identification.

**Figure 5:**
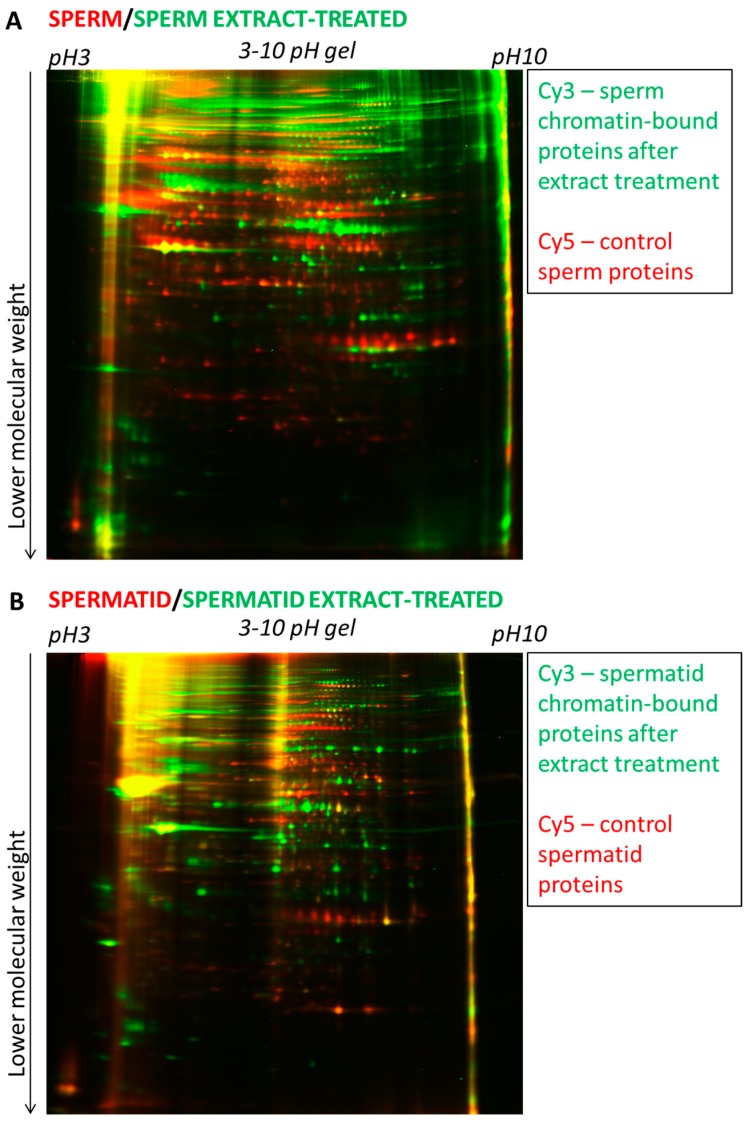
2D-DIGE electrophoresis of proteins from sperm and sperm-extract treated and of spermatid and spermatid-extract treated. (**A**) Proteins isolated from sperm (red) were run on 2-D gel together with proteins bound to the sperm chromatin after egg extract treatment (green); (**B**) Proteins isolated from spermatid (red) were run on 2-D gel together with proteins bound to the spermatid chromatin after egg extract treatment (green). Note the presence of numerous red or green spots on both gels (**A**,**B**) and the low number of yellow spots. The first dimension electrophoresis for both gels shown was carried in the pH 3–10 (broad range).

After the egg extract treatment of sperm and spermatid chromatin, the chromatin was isolated together with chromatin-bound proteins. Extensive washes (including centrifugation through a sucrose cushion) were used in order to enrich for proteins bound to chromatin (Figure 4 and Experimental Section). Proteins were then isolated, labelled with Cy3 and Cy5 dyes and subjected to 2D-DIGE analysis. Since only two samples can be simultaneously compared on one gel and there were four samples to be compared (sperm, sperm-extract treated, spermatid, spermatid-extract treated), we first compared sperm with sperm-extract treated and spermatid with spermatid-extract treated (Figure 5A,B, respectively). Interestingly, we observed that virtually all the detectable proteins changed after the egg extract treatment (almost no “yellow” spots, see Figure 5A,B). This can be explained by two possibilities: (1) the great majority of the donor nuclear proteins are removed (and/or modified post-translationally) upon the incubation with egg extract or (2) egg-derived proteins are in such excess over the donor cell-derived proteins that the latter ones become undetectable after the egg extract treatment.

#### 2.2.2. Many Egg Proteins Bind Differentially to Sperm and to Spermatid Chromatin

**Figure 6:**
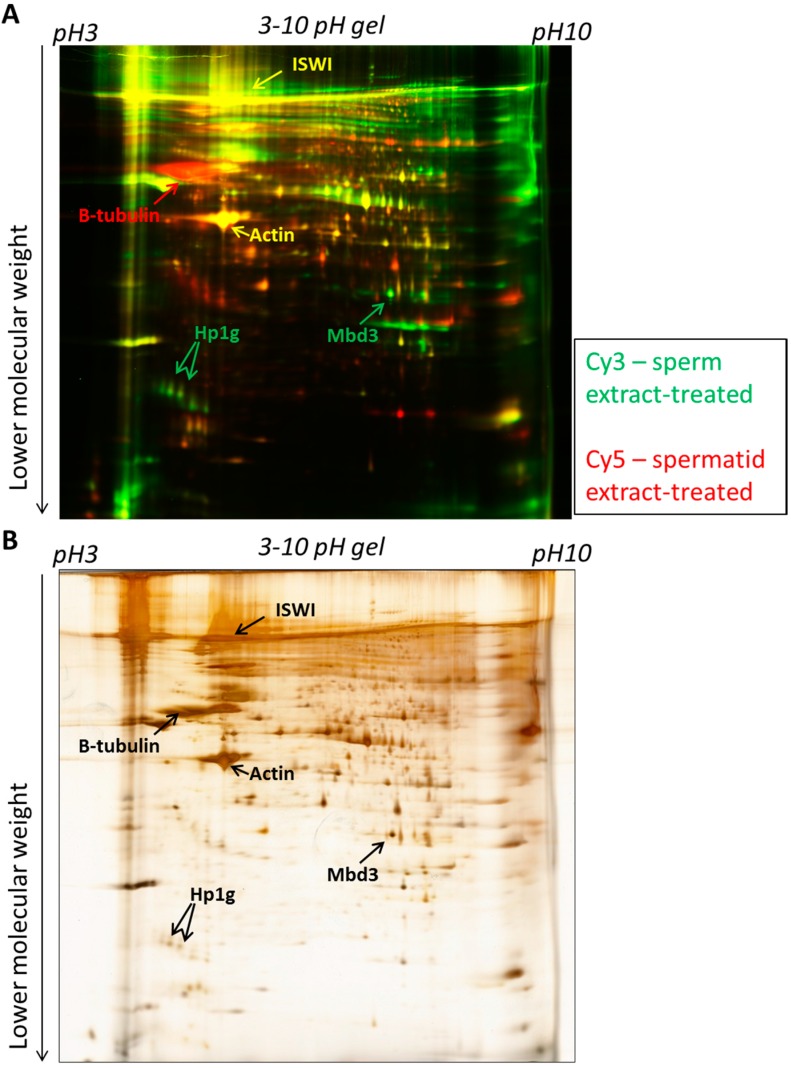
2D-DIGE electrophoresis of sperm-extract treated and spermatid-extract treated. (**A**) Proteins bound to sperm chromatin after egg extract treatment (green) were run on a pH 3–10 gel together with proteins bound to spermatid chromatin after egg extract treatment (red). Examples of proteins binding specifically to sperm (green), spermatid (red) or to both types of cells (yellow) are indicated with arrows; (**B**) The same gel as in (**A**) silver-stained to allow protein spot visualisation and excision. Examples of proteins are indicated with arrows.

Since we showed that almost all detectable proteins change after the egg extract treatment of sperm or spermatids, we performed a direct comparison of sperm-extract treated sample with the spermatid-extract treated sample to identify egg proteins that are specifically incorporated from extracts to the sperm or spermatid nuclei and also to reveal the identity of the protein incorporated into both cell types. 2D-DIGE analysis identified numerous protein spots present specifically in the sperm extract-treated or in the spermatids extract-treated sample, and also proteins binding into both chromatin types (Figure 6A). Selected spots were excised and subjected to mass spectrometry-based identification (Figure 6B). This led to the identification of 107 proteins bound specifically to sperm nuclei, 20 proteins bound to spermatid nuclei and 108 proteins incorporated from the egg into both cell types (Table S3).

#### 2.2.3. Known Chromatin Remodelling Factors and Replication Proteins Were Incorporated from Egg Extracts into both Sperm and Spermatids

Among the egg proteins that were bound to both types of nuclei, many proteins were involved in global chromatin remodelling. For example, chromodomain helicase DNA binding protein 4 (Chd4) and Imitation Switch (ISWI) were identified. Both Chd4 and ISWI are ATP-dependent chromatin remodelling complexes that move nucleosomes. Nucleosome sliding can change the accessibility of the DNA and plays important roles in multiple biological process, such as regulation of transcription mediated by RNA polymerases I-III, DNA replication or DNA repair [31,32,33]. ISWI has already been reported to be recruited to chromatin of somatic cell nuclei incubated in egg extracts [34], and hence its identification here validates the experimental setup applied.

Another class of proteins abundantly detected in both types of cells upon extract treatment are structural proteins that are important for the maintenance of the cell cytoskeleton and of the nucleus and nuclear structures, e.g., actin, nucleoporin or nuclear pore complex proteins Nup98-Nup96 [35,36,37,38], which likely reflect pronuclear formation activities induced by the extract. This is good agreement with previous studies [39,40,41].

An example of another highly represented group of proteins incorporated from the egg extract into sperm and spermatid nuclei are proteins directly involved in DNA replication: origin recognition proteins (Orc1), protein involved in unwinding and remodelling of the DNA (topoisomerase I and II, FACT (facilitates chromatin transcription) complex subunit Spt16, DNA replication helicase Mcm2), but also proteins involved in stabilizing the single stranded DNA, necessary for replication, such as Replication protein A (RPA) [42,43,44,45,46,47,48]. Identification of the types of proteins mentioned above confirms that the extracts used were functional and that they were able to recapitulate the events occurring in the first embryonic cell cycle, for example, DNA replication.

Last, a protein named Sal-like protein 4 (Sall4) was also identified as bound to both cell types after egg extract treatment. Sall4 was highly abundant among the proteins incorporated into chromatin after the egg extract treatment, as it was identified as the fourth most abundant protein in all the groups analysed (Table S3). Interestingly, Sall4 is a transcription factor and was reported as a master regulator of the core pluripotency network, necessary for the early embryonic development [49,50,51]. This finding implies that factors important for the pluripotency in the early embryo are incorporated into the paternal chromatin immediately after fertilization.

#### 2.2.4. Distinct Protein Isoforms Identified in Extract-Treated Sperm- and Spermatids

Interestingly, some proteins that were identified in spots originating uniquely from extract-treated sperm or from extract-treated spermatids, were identified as different isoforms sharing a high degree of similarity. For example, a protein identified within sperm-extract treated (gi|27881711) was just 3 amino acid different from protein identified in spermatids-extract treated (gi|639691) and both of them were isoforms of High mobility group protein X (HMG-X) [52]. Another such example is a protein named Hira (histone cell cycle regulation defective homolog A) [53]. One isoform of this protein (Hira-A) was identified as specific for sperm-extract treated (gi|50416397) and another one (Hira) as present in both cell types after egg extract treatment (gi|14330670). There were also some cases in which the same protein was identified separately in sperm- and spermatid-extract treated. For example, Metastasis associated 1-like protein (mta2) (gi|5901733) was identified independently in sperm- and in spermatid-specific protein spots. This is likely a result of post-translational modification of the protein; however, due to the fact that the analysis performed here did not discriminate between different post-translational modifications, such proteins were classified as present in both cell types after the egg extract treatment.

#### 2.2.5. Many Egg-Derived Chromatin Factors Bind Preferentially to Sperm Nuclei

Numerous interesting egg proteins were bound preferentially to one chromatin type and not to the other. Many of the proteins identified exclusively in sperm extract-treated were structural chromatin proteins, for example core histones: H2A, H2B, H3 and H4. Presence of core histones incorporated into the sperm chromatin likely reflects the remodelling of the paternal chromatin after fertilization and the exchange of sperm-derived protamine-like proteins (sp1–6) to a canonical type of histones derived from the egg.

Another class of egg proteins binding specifically to the sperm chromatin is chromatin-binding transcriptional repressors, such as heterochromatin protein 1 gamma (HP1γ), methyl-CpG binding domain protein 3 (Mbd3), histone deacetylase 1 and histone deacetylase 2 (Hdac1 and Hdac2, respectively). HP1γ was shown to recognize and to bind methylated lysine 9 of histone H3 [54]. This binding is important for the regulation of gene expression [55,56] and also for cell reprogramming to pluripotency [57]. Mbd3 does not recognize post-translational marks on histones, but binds to methylated DNA, and lead to transcriptional repression of the target genes [58]. Mbd3 was shown to be necessary for embryonic development in *Xenopus* [59] and to be important for the regulation of pluripotency [60,61]. Hdac1 and Hdac2 are enzymes responsible for removal of acetyl marks from histones. This Hdac-mediated deacetylation was shown to be involved in transcriptional repression [62,63]. Furthermore, both Hdac1 and Hdac2 are involved in DNA replication, for example by stabilizing newly formed nucleosomes and also by directly interacting with topoisomerase II [64,65]. Identification of many repressive egg proteins binding specifically to the sperm chromatin seems surprising. However, during the rapid cell cycle phases of early *Xenopus* development, no transcription is observed [66]. Therefore, the ability to recruit various repressive proteins from the egg may reflect programming of sperm to participate in the earliest phases of embryonic development—to support efficient replication and prevent premature transcription of the embryonic genome.

Such a wide variety of chromatin remodelling proteins was not identified in extract treated spermatids, with the exception of Baf57/Smarce1. Baf57 was shown to be important for cell cycle progression via transcriptional regulation of cell-cycle related genes [67]. Furthermore, a couple of unique isoforms of structural proteins—tubulin and vimentin were identified as binding specifically to spermatid extract-treated (Table S3).

#### 2.2.6. GO (Gene Ontology) Analysis Confirms the Overrepresentation of Egg Chromatin Remodelling Factors Binding Specifically to Sperm Nuclei

GO analysis of egg proteins binding specifically to sperm, to spermatids and to both cell types confirmed the observations made by looking at examples of proteins (Table S4). First, among the egg proteins incorporated specifically to the sperm chromatin, there was a significant overrepresentation of those responsible for chromosome and chromatin organization, chromatin modification, chromatin assembly and disassembly. Such terms were not enriched among proteins incorporated into spermatid-nuclei, and instead terms related to protein polymerization and protein complex assembly were identified as significantly enriched. As expected, many cell cycle related terms were enriched among egg proteins incorporated into both cell types, such as DNA replication, mitosis, cell division, spindle assembly *etc.* This likely reflects the functionality of the extracts used for the experiments, and their ability to support the events happening during the first embryonic cell cycle.

### 2.3. Validation of Mass Spectrometry Results by Immunoblotting

**Figure 7:**
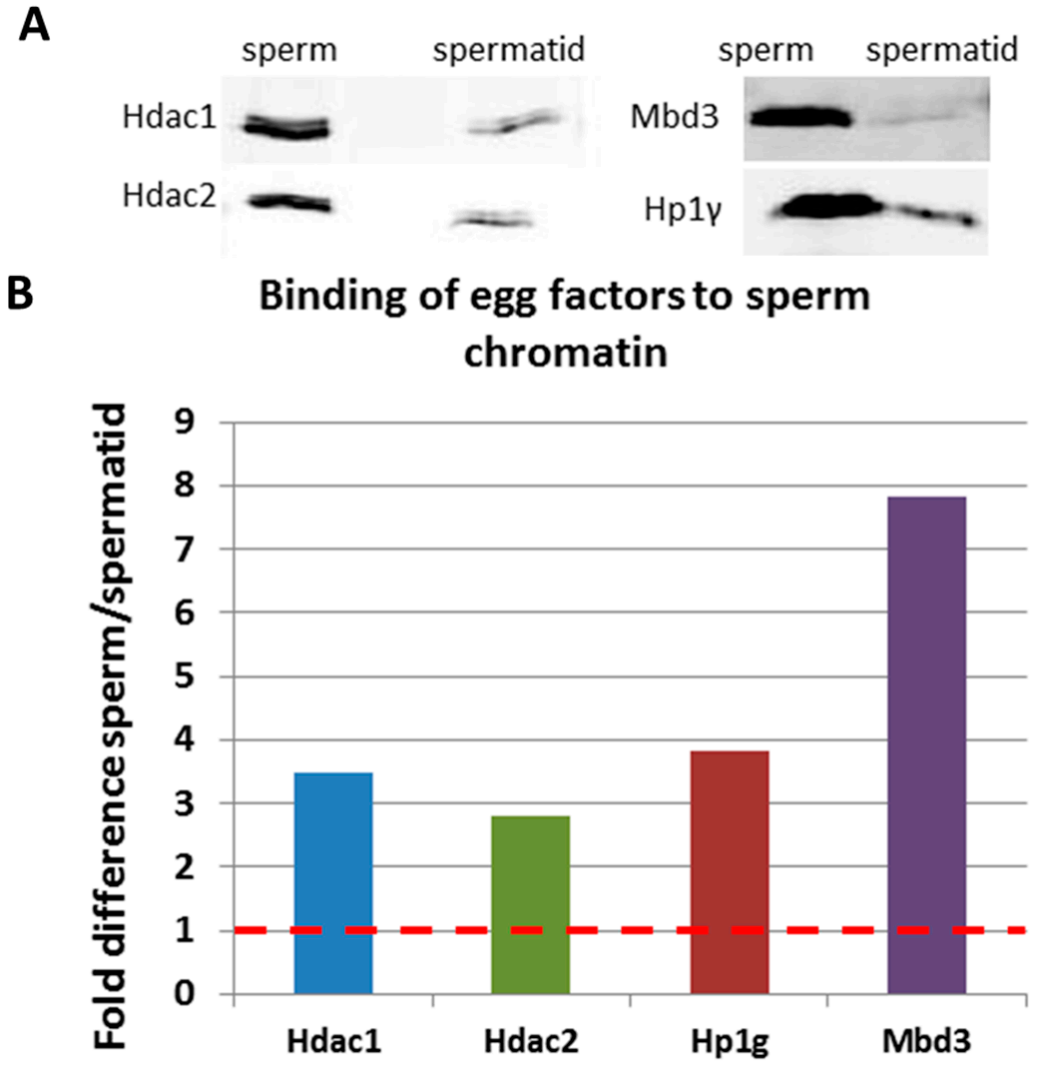
Validation of mass spectrometry results by immunoblotting. (**A**) Immunoblotting results on proteins isolated from sperm and spermatids after extract treatment. “sperm”—proteins bound to sperm chromatin after egg extract treatment; “spermatid”—proteins bound to spermatid chromatin after egg extract treatment. The antibody used is indicated on the left hand side of the blot inset. 6 µg of protein lysate was loaded on each lane; (**B**) Quantification of the blots shown in (**A**). Results are shown as fold difference between the band intensity in sperm-extract treated to spermatid extract-treated. Names of the proteins are indicated below the *x* axis.

To validate the mass spectrometry results we performed immunoblotting analysis for candidate factors identified by mass-spectrometry analysis. We have chosen four proteins, Hdac1, Hdac2, Hp1γ and Mbd3, identified as binding preferentially from the egg to the sperm chromatin. These particular proteins were chosen due to the availability of antibodies that recognize the *Xenopus laevis* proteins. Immunoblotting analysis confirmed that those proteins were preferentially incorporated into the sperm chromatin upon egg extract treatment (Figure 7A,B), therefore validating the use of mass spectrometry approach.

## 3. Experimental Section

### 3.1. Isolation and Permeabilization of Sperm and Spermatids

Sperm and spermatids were isolated using density gradient centrifugation method, adapted from [13] with a minor modification: iodixanol (Sigma, Dorset, UK; D1556) gradients were used instead of metrizamide. Following centrifugation, sperm (pellets), and partially purified spermatids (at the interface of the iodixanol solutions), were collected. Throughout the manuscript these two partially purified fractions are called sperm and spermatid, respectively. Cells were subsequently permeabilized with Digitonin (10 mg/mL as a final concentration) for 5 min at room temperature as detailed before [68].

### 3.2. Isolation of Proteins and Sample Preparation for 2D-DIGE

Proteins from pelleted sperm and spermatids were extracted in Urea/Thiourea buffer (4% Chaps, 2 M Thiourea, 6 M Urea; Sigma: C9426, T7875, respectively and Fisher Chemicals, Loughborough, UK; U/0500/53) supplemented with protease inhibitors (Roche, Welwyn Garden City, UK; 11873580001) by keeping on ice for 1 h and vortexing from time to time until the pellet disappeared. Subsequently, the solution was sonicated with a probe sonicator (Diagenode, Liege, Belgium) (3 s, Amplitude 40, sonication over an ice bath) in four separate rounds. The protein concentration was quantified with a Quick Start Bradford Protein Assay (Bio-Rad, Hemel Hempstead, UK; 500-0202), following manufacturer’s recommendations. Fifty micrograms of protein lysate in 10 µL volume was subsequently labelled with Cy3 or Cy5 dye (GE Healthcare, Hatfield, UK) (Cy dyes label the lysine residues of proteins) by adding 1 µL of 0.2 mM dye. Labelling reaction was carried at 4 °C for 30 min in the dark and quenched by the addition of 1 µL of 1 mM lysine. Quenching was performed for 10 min at 4 °C in the dark. After the labelling was completed, labelled protein lysates were mixed. Reciprocal labelling was performed to rule out any abnormalities or biases in labelling.

### 3.3. 2D-DIGE Analysis

Compared samples were mixed and loaded together on 7–11 NL (non linear), 13 cm or 3–10 NL, 13 cm immobiline strip (GE Healthcare) using cup loading method for 7–11 pH range and rehydration loading for 3–10 NL strip, according to the manufacturer’s protocol. The proteins were focused on the strip using IPGphor isoelectric focusing system for a total of 52,000 VHrs and separated by second dimension electrophoresis on 12% Sodium Dodecyl Sulfate (SDS) polyacrylamide gels using SE 600 vertical unit (GE Healthcare). Gels were scanned on Typhoon 9400 laser scanner (GE Healthcare). The images showed clear, visual differences between compared samples which were again confirmed by reciprocal labelling. For qualitative analysis of differently expressed proteins gels were silver stained according to [69] and spots of interest excised for further mass spectrometry analysis.

### 3.4. Spot Excision and Protein Preparation for Mass Spectrometry Analysis

2D gel spots were excised with a 10 mL piptette tip and transferred into a 96-well PCR plate. The gel spots were then destained, reduced with dithiothreitol (DTT), alkylated with iodoacetamide and subjected to enzymatic digestion with trypsin overnight at 37 °C. After digestion, the supernatant was pipetted into a sample vial and loaded onto an autosampler for automated LC-MS/MS analysis.

### 3.5. LC-MS/MS Analysis and Protein Identification

All LC-MS/MS experiments were performed using a nanoAcquity UPLC (Waters Corp., Milford, MA, USA) system and an LTQ Orbitrap Velos hybrid ion trap mass spectrometer (Thermo Scientific, Waltham, MA, USA). Separation of peptides was performed by reverse-phase chromatography using at a flow rate of 300 NL/min and a Waters reverse-phase nano column (BEH C18, 75 µm i.d. × 250 mm, 1.7 µm particle size). Peptides were loaded onto a pre-column (Waters UPLC Trap Symmetry C18, 180 µm i.d. × 20 mm, 5 µm particle size) from the nanoAcquity sample manager with 0.1% formic acid for 3 min at a flow rate of 10 µL/min. After this period, the column valve was switched to allow elution of peptides from the pre-column onto the analytical column. Solvent A was water +0.1% formic acid and solvent B was acetonitrile +0.1% formic acid. The linear gradient employed was 5%–50% B in 25 min and the total run time was 40 min.

The LC eluant was sprayed into the mass spectrometer by means of a New Objective nanospray source. All *m*/*z* values of eluting ions were measured in an Orbitrap Velos mass analyzer (Thermo Fischer Scientific, Waltham, MA, USA), set at a resolution of 30,000. Data dependent scans (Top 20) were employed to automatically isolate and generate fragment ions by collision-induced dissociation in the linear ion trap, resulting in the generation of MS/MS spectra. Ions with charge states of 2+ and above were selected for fragmentation. Post-run, the data was processed using Protein Discoverer (version 1.2., Thermo Fischer Scientific). Briefly, all MS/MS data were converted to mgf files and these files were then submitted to the Mascot [17] search algorithm (Matrix Science, London, UK) and searched against a custom *Xenopus* database, containing NCBI entries from *Xenopus laevis* and *Xenopus tropicalis*, using a fixed modification of carbamidomethyl (C) and a variable modification of oxidation (M). Peptide identifications were accepted if they could be established at greater than 95.0% probability.

### 3.6. Interphase Egg Extract Preparation

Eggs were collected in 1× MMR (Marc’s modified Ringer’s), dejellied with 0.2× MBS (Modified Barth’s Saline), 2% cysteine (pH 7.8–7.9) (Sigma, W326305) and washed with 0.2× MMR. Subsequently, eggs were activated for 3 min at room temperature (RT) with 0.2× MMR supplemented with 0.2 µg/mL calcium ionophore (Sigma, C7522). Eggs were rinsed with 0.2× MMR and subsequently all abnormal or not activated eggs were removed. Eggs were washed with 50 mL of ice-cold extraction buffer (EB) (5 mM KCl, 0.5 mM MgCl_2_, 0.2 mM DTT, 5 mM Hepes pH 7.5) supplemented with protease inhibtiors (PI) (Roche, 11873580001), transferred into centrifugation tube (Thinwall, Ultra-Clear™, 5 mL, 13 × 51 mm tubes, Beckman Coulter Inc., High Wycombe, UK; 344057) and supplemented with 1 mL of EB buffer with PI and 100 µg/mL of cytochalasin B (Sigma, C2743) and placed on ice for 10 min. Subsequently, eggs were spun briefly at 350× *g* for 1 min at 4 °C (SW55Ti rotor, Beckman Coulter Ultra-centrifuge, Optima L-100XP) and excess buffer was discarded. Eggs were then spun at 18,000× *g* for 10 min at 1 °C, the extract was collected with a needle, transferred to a fresh, pre-chilled tube, supplemented with PI and 10 µg/mL of cytochalasin B and re-spun using the same conditions. Extract was collected with a needle and used: frozen (on liquid nitrogen, in 100 µL aliquots) for the analysis of proteins bound to the chromatin followed by the proteomic analysis; or fresh, for replication assays (see below).

### 3.7. Egg Extract Treatment and Protein Isolation for Mass Spectrometry Analysis of Egg Proteins Incorporated into Sperm or Spermatid Chromatin

Sperm and spermatids were collected and permeabilised as described above. Egg extracts were prepared as described above. Twenty million permeabilised sperm or spermatids were treated with 3650 µL of egg extract, supplemented with the 1× final energy regeneration mix (20× energy regeneration mix is prepared and stored in aliquots at −80 °C: 2 mg/mL Creatine Kinase (Roche, 10127566001), 150 mM Creatine Phosphate (Roche, 10621714001), 20 mM ATP (Roche, 10519979001), 2 mM ethylene glycol tetraacetic acid (EGTA), 20 mM MgCl_2_). Control permeabilised cells were treated with EB buffer and the energy regeneration mix alone. Cells were incubated in the extract/buffer for 1 h at room temperature with frequent tapping. After that, cells were washed with 15 volumes of Buffer D (10 mM Hepes pH 7.7, 10 mM KCl, 1.5 mM MgCl_2_, 1 mM DTT) (spin at 3220 rcf, 4 °C, 20 min). Pellets of black colour were observed in the egg extract-treated samples. Subsequently, pellets were resuspended in 15 mL of Buffer E (250 mM Sucrose, 10 mM Tris-HCl pH 8.0, 5 mM MgCl_2_, 1 mM DTT, 0.1% Triton X-100) and spun at 3220 rcf, 4 °C, 20 min. Subsequently, pellets were resuspended in 1 mL of Buffer E each and transferred to a 1.5 mL tubes and washed 6 more times with Buffer E (each time spun at 1000 rcf, 5 min, 4 °C). The final pellet contained proteins bound to chromatin, which were isolated and processed for 2D-DIGE and proteomics analysis as described in Section 2.5.

### 3.8. Immunoblotting Analysis

All the immunoblotting analyses were performed according to standard immunoblotting protocols [70]. For the validation of mass spectrometry results, equal amount of proteins isolated in Urea/Thiourea buffer were loaded on each blot. Polyacrylamide gels were cast at the percentage appropriate for the separation of the desired protein size [70]. Gel electrophoresis, transfer of proteins, immunoblotting and washes were performed according to the standard protocols [70]. Polyvinylidene difluoride (PVDF) membranes with 0.45 µM pore size were used (Immobilon, Millipore, Watford, UK; IPFL00010) with a semi-dry transfer system (Trans-Blot^®^ SD Semi-Dry Transfer Cell, BioRad, Hemel Hempstead, UK; 170-3940) transferring for 30 min, at room temperature (25 V). All the protein detections were performed using immunofluorescence with the use of the LI-COR Imaging System. Primary antibodies were used at 1:1000 dilutions, unless stated otherwise and blots were incubated with the primary antibodies overnight at 4 °C. Primary antibodies against the following proteins were used in this thesis: Hdac1 (rabbit polyclonal; Abcam, Cambridge, UK; ab33278), Hdac2 (rabbit polyclonal; Epitomics, Burlingame, CA, USA; S2398), Mbd3 (1:250; mouse monoclonal; Abcam, ab45027), Hp1γ (goat polyclonal; Abcam, ab40827), Rbbp4 (rabbit polyclonal; Abcam, ab1765), Rbbp7 (rabbit polyclonal; Abcam, ab3535) and Lsf (rabbit polyclonal; Abcam, ab80445; To detect the primary antibodies, the following secondary antibodies were used (all at 1:25,000 dilution): goat anti-mouse IRDye 800 (LI-COR Biosciences, Cambridge, UK; 926-32210), Goat anti-Rabbit IRDye 800 (LI-COR Biosciences;926-32211), goat anti-rabbit Alexa 680 (Invitrogen, Paisley, UK; A-21109), goat anti-mouse Alexa 680 (Invitrogen, Paisley, UK; A-21057), donkey anti-goat Alexa 680 (Invitrogen, Paisley, UK; A-21084). Blots were incubated with the secondary antibody solution for 1 h at RT. To reveal the proteins, blots were scanned using a LI-COR detection system (Odyssey laser scanner, LI-COR Biosciences).

## 4. Conclusions

To conclude, the mass spectrometry analysis of proteins differentially expressed in sperm, spermatids and in egg extract-treated sperm and spermatids allowed the identification of numerous proteins present specifically in each of these cell types. Some of these proteins were previously identified as present in similar samples, therefore validating the approach used, for example the presence of Sp1 basic protein in sperm, Rsb-66 in spermatids or the incorporation of imitation switch (ISWI) into both types of nuclei after the egg extract treatment. On the other hand, our approach also enabled us to make interesting novel findings. For example, Wdr5 protein was found exclusively in sperm. Since Wdr5 was previously implicated in regulation of transcription, its presence in sperm, but not in spermatid, may contribute to sperm programming to support efficient embryonic transcription. Also, several proteins, like Hp1γ, were identified as incorporated specifically to the sperm chromatin after the egg extract treatment. This could reflect interplay between the sperm chromatin and the egg’s cytoplasm; unique features of sperm, but not of spermatid chromatin, may allow the binding of specific egg factors, such as Hp1γ.

It is also important to note that the approach used here to identify the proteins has some limitations. First, proteins need to be sufficiently abundant in order to be identified by 2D-DIGE/mass spectrometry approach. Second, due to technical limitations only some of the spots were excised and analysed by LC-MS/MS. This means that not all the proteins that are different between sperm and spermatids and not all the egg proteins preferentially binding to the sperm or spermatid chromatin were identified here. Nevertheless, our results clearly show that the 2D-DIGE approach coupled with LC-MS/MS is a powerful tool for screening for the most robust differences between the samples, as it allowed us to identify novel interesting proteins that could be involved in sperm programming for embryonic development. Perhaps the most interesting and unexpected finding is the identification of many transcriptional repressors, such as Mbd3, Hp1γ or Hdac1, as egg factors binding specifically to the sperm chromatin. This suggests that these factors could be important for the maintenance of transcriptional silencing during rapid cell cycle divisions in early *Xenopus* development. In future, it will be interesting to test the functional importance of these factors, and to test whether their incorporation into sperm chromatin at fertilization is indeed required for correct embryonic development.

## Supplementary Materials

Supplementary materials can be found at: http://www.mdpi.com/1422-0067/15/19/16719/s1.

## Supplementary Files

Supplementary File 1
